# Impact of increased ventilation on indoor temperature and malaria mosquito density: an experimental study in The Gambia

**DOI:** 10.1098/rsif.2020.1030

**Published:** 2021-05-12

**Authors:** Ebrima Jatta, Majo Carrasco-Tenezaca, Musa Jawara, John Bradley, Sainey Ceesay, Umberto D'Alessandro, David Jeffries, Balla Kandeh, Daniel Sang-Hoon Lee, Margaret Pinder, Anne L. Wilson, Jakob Knudsen, Steve W. Lindsay

**Affiliations:** ^1^ National Malaria Control Programme, Banjul, The Gambia; ^2^ Department of Biosciences, Durham University, Durham, UK; ^3^ Medical Research Council Unit The Gambia at the London School of Hygiene & Tropical Medicine, Banjul, The Gambia; ^4^ London School of Hygiene & Tropical Medicine, London, UK; ^5^ Royal Danish Academy - Architecture, Design, Conservation, Copenhagen, Denmark; ^6^ Liverpool School of Tropical Medicine, Liverpool, UK

**Keywords:** housing, malaria, ventilation, carbon dioxide, human comfort, sub-Saharan Africa

## Abstract

In sub-Saharan Africa, cooler houses would increase the coverage of insecticide-treated bednets, the primary malaria control tool. We examined whether improved ventilation, using windows screened with netting, cools houses at night and reduces malaria mosquito house entry in The Gambia. Identical houses were constructed, with badly fitting doors the only mosquito entry points. Two men slept in each house and mosquitoes captured using light traps. First, temperature and mosquito density were compared in four houses with 0, 1, 2 and 3 screened windows. Second, carbon dioxide (CO_2_), a major mosquito attractant, was measured in houses with (i) no windows, (ii) screened windows and (iii) screened windows and screened doors. Computational fluid dynamic modelling captured the spatial movement of CO_2_. Increasing ventilation made houses cooler, more comfortable and reduced malaria mosquito house entry; with three windows reducing mosquito densities by 95% (95%CI = 90–98%). Screened windows and doors reduced the indoor temperature by 0.6°C (95%CI = 0.5–0.7°C), indoor CO_2_ concentrations by 31% between 21.00 and 00.00 h and malaria mosquito entry by 76% (95%CI = 69–82%). Modelling shows screening reduces CO_2_ plumes from houses. Under our experimental conditions, cross-ventilation not only reduced indoor temperature, but reduced the density of house-entering malaria mosquitoes, by weakening CO_2_ plumes emanating from houses.

## 1. Introduction

Between 2000 and 2015, massive deployment of malaria control interventions in sub-Saharan Africa reduced malaria prevalence by half and clinical malaria by 40% [1]. Of the interventions used to achieve this remarkable level of control, insecticide-treated nets (ITNs) were widely used and most effective, contributing to 68% of the reduction in malaria infection prevalence. The World Health Organization's target of universal coverage with ITNs requires the distribution of one bednet for two people at risk of malaria, repeated every three years [2]. In 2019, although 68% of households in sub-Saharan Africa had at least one net, only 46% of people reported using a net [3], mainly because it was too hot to sleep under a net [4]. Cooling a house at night, by improving ventilation, could increase net use and further reduce the malaria burden.

*Anopheles gambiae* s.l., the principal vectors of malaria in sub-Saharan Africa, bite predominantly indoors at night [5]. These mosquitoes locate a blood meal using a range of chemical cues generated by people, particularly carbon dioxide (CO_2_) [6]. This gas is a major component of exhaled breath and stimulates take-off, extends flight duration [7] and is a long-range attractant for *An. gambiae* and other mosquitoes [6,8]. Thus to understand the role of ventilation in a building, one needs to understand how this gas accumulates in inhabited rooms and leaks out of buildings, since the concentration and shape of the odour plumes will affect how readily mosquitoes are able to locate people within a house.

One simple way to ventilate a bedroom, and cool the room at night, is to install mosquito screening on doors and windows [9]. We designed a series of experiments to explore the effect of house screening on indoor temperature and mosquito house entry and used computational fluid dynamic (CFD) modelling [10] to visualize the distribution of the CO_2_ and to provide realistic approximations of CO_2_ concentrations generated by people sleeping in single-roomed houses with and without mosquito screening. Our findings are relevant to those interested in designing and constructing buildings that protect people from malaria in sub-Saharan Africa.

## 2. Methods

### 2.1. Study design

**Figure 1.**
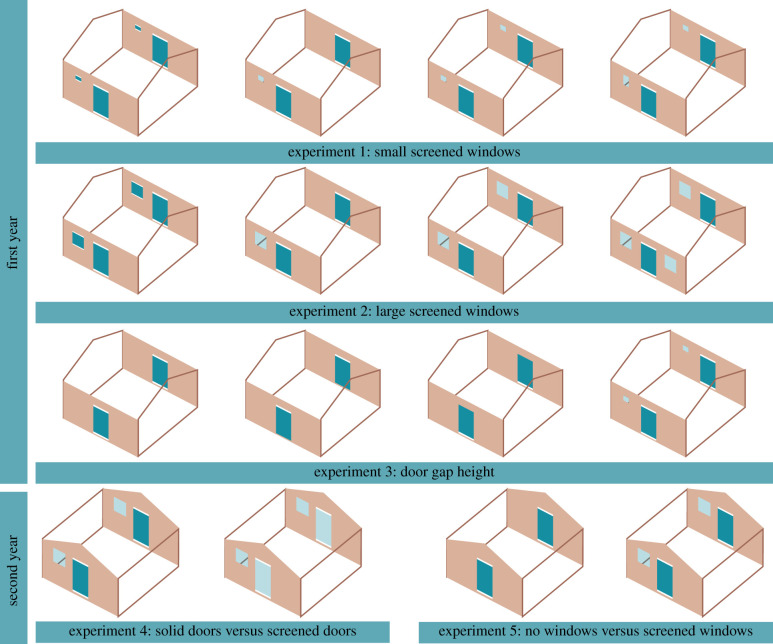
Summary of experiments. The reference house in each experiment is shown in the first column of each row. In the experiments, local, badly fitting doors and windows were mimicked by adding narrow gaps above and below the doors and some windows. Screened windows are shown as translucent squares and rectangles.

A detailed description of the experiments is provided in the electronic supplementary material. Briefly, in year 1, four square single-roomed experimental houses constructed with mud walls, metal roofs and closed eaves, each sleeping two men, were used to examine the effect of insecticide-free screened windows on indoor climate and mosquito house entry (figure 1). In all experiments, each house had narrow slits above and below two metal doors, which constituted the main entry point for mosquitoes, and represented badly fitting doors, commonly found in local villages. We carried out three experiments in year 1. Experiment 1 compared houses with badly fitting screens (control) to houses with one small-screened window, two small-screened windows, one small-screened window and one medium-screened window. Experiment 2 maintained the same control compared to houses with one, two or three large-screened windows. Experiment 3 compared unscreened houses with narrow slits above and below the doors with those with slits above or below the door. In each house, temperature and relative humidity were measured using a data logger and mosquitoes captured using a CDC light trap. Collections were made over 25 nights and typologies rotated between houses each week, so that at the end of each experiment, each typology had been tested in each house.

We found that increasing ventilation, by increasing the number and size of windows, had a small cooling effect, but, surprisingly, also markedly reduced the numbers of malaria mosquitoes entering the house. We formulated three hypotheses to explain why screened windows reduced mosquito house entry through the slits above and below the doors. These were that screening: (i) acts as a site of attraction, with mosquitoes attracted to host odours leaking from the screening, accumulating on the outside of the window screening; (ii) reduces indoor concentrations of host odours by a combination of increased ventilation and a cooler house reducing sweating and the production of host odours; and (iii) changes the shape of odour plumes emanating from a house, attracting fewer mosquitoes.

To test these hypotheses, we carried out a further series of field studies using two experimental houses in year 2, repeating the basic study design from year 1, but, in addition to routine monitoring, also measured indoor CO_2_ concentrations. In experiment 4, one house with solid doors was compared with one with screened doors, both doors having narrow slits top and bottom. If hypothesis 1 was correct, more mosquitoes would be collected in the house with screened doors than solid doors, since mosquitoes accumulating outside the screened door, attracted to odours from the door, would enter the house through the door gaps in larger numbers than houses with solid doors. In experiment 5, both houses had solid doors, one had screened windows and one had no windows. This experiment allowed us to confirm the findings of experiments 1 and 2, but in addition allowed us to measure indoor CO_2_ concentrations to test hypotheses two and three. Typologies were rotated between houses weekly and each experiment lasted 20 nights.

### 2.2. Study area

In year 1, in 2017, the study took place outside Wellingara village (N 13°33.365′, W 14°55.461′), adjacent to a large area of irrigated rice, and, in year 2, in 2019, it took place approximately 2 km to the north, in the grounds of the Medical Research Council's field station at Wali Kunda (N 13°34.25′, W14° 55.28′). Both sites are situated on the south bank of the River Gambia, in the Central River Region. The study was done during the rainy season, from June to October, when malaria transmission is high and people are most likely to sleep under an ITN [11].

### 2.3. Experimental houses

In year 1, experiments were conducted from 2 July to 23 October 2017. Construction of the experimental houses has been described in detail elsewhere [12]. Briefly, four square single-roomed mud-block houses were constructed on the western edge of Wellingara village and two in Wali Kunda field station. Each house was the average size of single-roomed houses in the Upper River Region and was built along a line, 10 m apart. Each house was 4.20 m by 4.20 m in floor area, with walls 2.20 m high and a saddle-shaped roof, made of corrugate sheeting, with closed eaves. Each house had two doors (1.75 m high × 0.75 m wide), built on opposite sides of the house. In all experiments (apart from experiment 3), screened and unscreened doors had narrow slits, 0.02 m high and 0.75 m wide, above and below the door to simulate badly fitted doors common in local villages (electronic supplementary material, figure S1). In experiments 1 and 2, the reference house was a metal-roofed house which had narrow slits above and below the solid metal doors and windows (figure 1). In experiment 3, the reference house had no windows, only badly fitting doors. Polyester netting (2 × 2 mm mesh) screens mounted on metal frames were used for screening windows.

In year 2, field experiments were conducted during the rainy season, from 28 August to 11 November 2019. Experiments were conducted using two experimental houses of a similar design to those used in year 1, apart from the saddle roof apex in year 2 houses faced the front and back facades, rather than the sides, i.e. the roof was rotated 90° and the roofs had been painted red in year 2, rather than bare metal in year 1. We compared mosquito entry in screened and unscreened houses. As described earlier, two men slept in separate beds, under ITNs during the night and mosquitoes collected using light traps. We measured indoor and outdoor climate as before and also measured CO_2_ concentrations in the houses, to inform the modelling of CO_2_ movement within and outside the houses. At the start of each experiment, each typology was randomly allocated to one house position. Each house was arranged with two single beds located parallel to one another on opposite walls to the doors. Two adult men slept on separate beds in each house under an ITN (Olyset, Sumitomo Chemical, Japan) from 21.00 h to 06.00 h. The men remained in the house in the same position throughout each experiment, so that the relative attractiveness of the house positions to mosquitoes was combined with that of the pair of sleepers. For each experiment, CDC light traps were used to collect mosquitoes indoors for five weeks, each week for five nights. Each house typology was rotated weekly between houses using a replicated Latin rectangle design.

### 2.4. Procedures

In both years, mosquitoes were collected from each house using a CDC light trap (Model 512, John W. Hock Co., Gainesville, USA), sampled nightly for five nights each week for five weeks. Traps were suspended from the roof with the light 1 m above the ground in the centre of the room between the two sleepers at the foot end of the bed and operated from 21.00 h to 06.00 h. Mosquitoes were collected from each house at 06.00 h and killed by freezing. Mosquitoes were identified morphologically and female *An. gambiae* s.l. identified to species by PCR [13,14].

Indoor temperature and relative humidity were measured every 30 min using data loggers (Tiny tag, TGU 4500, Gemini Data Loggers, Chichester, UK) suspended 1 m from the floor in the centre of the room. In year 2, CO_2_ concentrations were recorded outdoors with the logger situated on a tripod at a height of 1.3 m, midway between the two experimental houses in Wali Kunda during the rainy season, from 2 July to 25 July 2017. Recordings were made on 12 nights from 21.00 to 06.59 h.

### 2.5. Outcomes

Primary outcomes were the mean indoor temperature, and the mean number of *An. gambiae* s.l./light trap/night for each house typology. Night-time temperature was analysed from 21.00 to 23.59 h, when adults in the study area go to bed and make a decision to sleep under a bednet or not [15], and from 00.00 to 06.59 h, when most are asleep.

### 2.6. Data analyses

Sample size calculations were based on mosquito counts since these are considerably more variable than the environmental recordings made. In year 1, the sample size was estimated via simulation based on data from a similar trial conducted in the study area, [16] where the mean number of *An. gambiae* s.l. collected indoors was 10.8/hut/night (standard deviation, s.d. = 8.7). The study was powered to detect an effect size of 50% at the 5% level of significance and 80% power and required 25 nights of observation. In year 2, we based our calculations on a study conducted in 2017 in nearby Wellingara village [12]. In this study, there was a mean of 6.2 *An. gambiae*/night (s.d. = 5.1). To detect 66% fewer mosquitoes indoors at the 5% level of significance, with 80% power, required 20 nights of observation.

The effect of house typology on indoor climate and mosquito house entry was assessed using generalized linear modelling, using a negative binomial model with a log link function for mosquito count data, while comparisons of indoor temperatures and CO_2_ were made using linear regression. In addition to house typology, house position and day were included in the model as fixed effects. For analysis of temperature and CO_2_ we calculated mean values for two separate periods, from 21.00 h to 23.30 h and 00.00 h to 07.00 h, for each house each night.

LadyBug software (LadyBug Products, Athol, ID, USA) was used to estimate the percentage of time occupants of various house typologies spent in the ‘comfort zone’ [17]. The human comfort index is widely used by those working in the built environment to assess how comfortable a building is and is based on experiments with hundreds of volunteers of different genders, ages and ethnicity wearing different amounts of clothing [18]. The index is used in the ANSI/ASHRAE Standard 55: Thermal Environmental Conditions for Human Occupancy. It is an American National Standard published by ASHRAE that establishes the ranges of indoor environmental conditions to achieve acceptable thermal comfort for occupants of buildings [19].

χ^2^-test was used to look for trends in human comfort with increasing numbers of large-screened windows and for comparisons between typologies. Analyses, apart from psychrometric analysis, were done using Stata version 16 (StataCorp, College Station, TX, USA), except for chi-square calculations which were done with Epi Info v. 3.01 (CDC, Atlanta, USA).

CFD modelling was used to simulate CO_2_ concentrations using Ansys^®^ Fluent (v. 19). Model assumptions and set-up configurations were as follows: (i) the house was based on the structure of an experimental house and was assumed air-tight, except for gaps at the top and bottom of the doors, and the screened doors and windows, (ii) two men were modelled as geometrically simplified mannequins with rectilinear body shapes, (iii) exhalation velocity of the mannequins was 0.77 m s^−1^ upwards, with 40 000 ppm of CO_2_, (iv) the temperature of exhaled breath was 32.85°C and body temperature 35.85°C, (v) from field data, background CO_2_ ppm was 410 ppm, and outdoor air temperature 25.5°C, (vi) outdoor night wind speed was 0.44 m s^−1^ and wind direction 54.3°, (vii) bednets and screens reduced air flow by 64%, (viii) air is incompressible, and air flow is steady turbulent flow, (ix) we used realizable *k–ε* turbulent models with enhanced wall treatments since they best matched field data and (x) the entire model had 7.5 million polyhedral cells, with 1 mm cells for the mouths and 10 mm for the nets.

CFD simulations were verified: (i) against detailed field data collected in Tanzania during the rainy season (D. Sang-Hoon Lee *et al*. in preparation) and (ii) using CO_2_ data logger recordings made in The Gambia. These simulations provide an accurate approximation of the site conditions at Wali Kunda with a 9% discrepancy.

### 2.7. Role of the funding source

The funders had no role in study design, data collection, data analysis, data interpretation or writing of the report. All authors had full access to all data in the studies and had final responsibility for the decision to submit for publication.

## 3. Results

### 3.1. Experimental houses

In year 1, there were roughly equal numbers of *An. arabiensis* (50.7%) and *An. coluzzii* (49.3%), while in year 2, *An. coluzzii* (69.0%) was more common than *An. arabiensis* (30.5%; electronic supplementary material, table S1). There were no differences in how each species responded to the different housing typologies.

**Figure 2.**
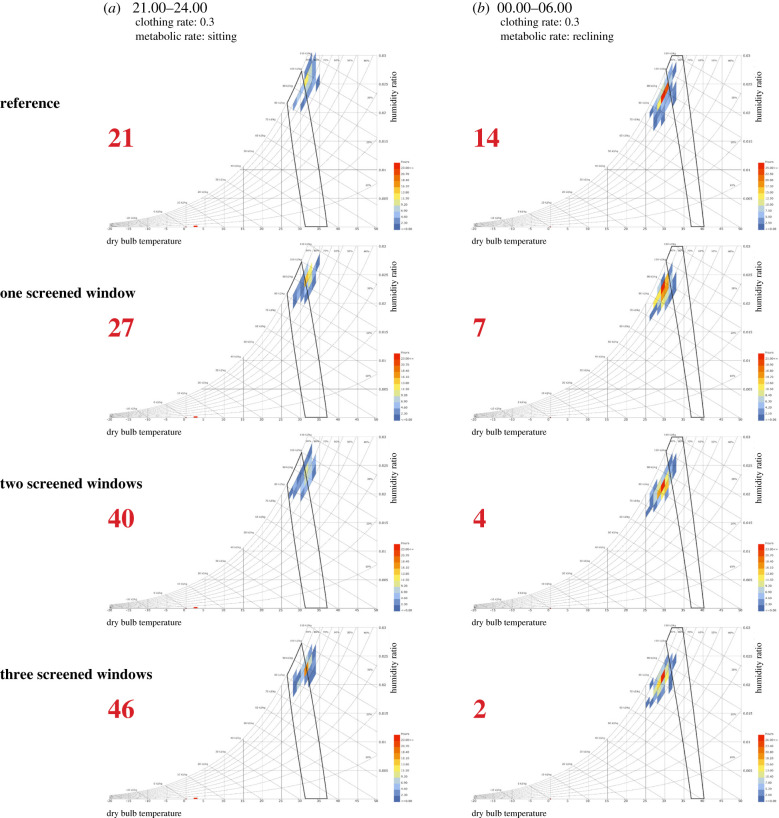
Psychrometric charts showing the human comfort index of adults in houses with and without large-screened windows. (*a*) Readings, shown as coloured polygons, made from 21.00 to 23.59 h. (*b*) Readings made from 00.00–06.00 h. Each data point represents a combination of temperature and relative humidity at different times of the night. Data points that fall within the black polygons represent values that are known to be comfortable for lightly dressed adults sitting or reclining. Values in red, percentage of readings comfortable.

In experiment 1, installing small-screened windows did not cool houses and were associated with an increased mean temperature of 0.2°C, both before and after midnight, compared to the reference house with badly fitting small windows (table 1, *p* < 0.001). By contrast, in experiment 2, installing two large-screened windows lowered indoor temperature by 0.4 to 0.5°C, and three large-screened windows by 0.6°C (*p* < 0.001) compared with the reference house with badly fitting large windows. The psychrometric analysis of large-screened windows in experiment 2 shows that from 21.00 to 23.59 h, human comfort increased with the increasing number of screened windows (*p* = 0.009), while from 00.00 to 05.59 h human comfort decreased with increasing numbers of windows since it became too cold (*p* = 0.0007, figure 2). Human comfort, however, was statistically different from the reference house only when there were two or more large-screened windows on opposite walls (21.00–23.59 h, *χ*^2^ = 3.9, *p* = 0.048; 00.00–05.59 h, *p* = 0.009).

**Table 1.** Indoor temperatures in year 1. CI, confidence intervals. General linearized modelling results, adjusting for house position and night. Temperatures of test houses are statistically significant from the reference house for both experiments and time periods (*p* < 0.001). Data are means (95% CIs). Larger drawings of the house typologies are shown in figure 1.

typology		period					
		21.00 to 23.59 h (*n* = 25)			00.00 to 06.00 h (*n* = 25)		
		mean temperature (°C)	temperature difference (°C)	*p*	mean temperature (°C)	temperature difference (°C)	*p*
experiment 1: small-screened windows							
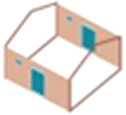	two badly fitting small windows (reference)	31.7 (31.6 to 31.8)	—	—	(29.4 to 29.6)	—	—
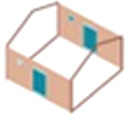	one small-screened window	31.9 (31.8 to 32.0)	0.19 (0.05 to 0.33)	<0.001	29.7 (29.6 to 29.8)	0.17 (0.05 to 0.28)	<0.001
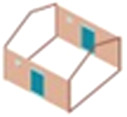	two small-screened windows	31.9 (31.8 to 32.0)	0.21 (0.07 to 0.35)	<0.001	29.7 (29.6 to 29.8)	0.18 (0.07 to 0.29)	<0.001
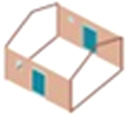	one small-screened window and one medium-screened window	31.9 (31.8 to 32.0)	0.21 (0.07 to 0.35)	<0.001	29.7 (29.6 to 29.8)	0.17 (0.06 to 0.29)	<0.001
experiment 2: large-screened windows							
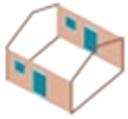	two badly fitting large windows (reference)	31.7 (31.6 to 31.8)	—	—	29.6 (29.5 to 29.6)	—	—
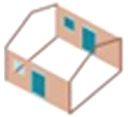	one large-screened window	31.6 (31.5 to 31.7)	−0.11 (−0.20 to −0.02)	<0.001	29.5 (29.4 to 29.5)	−0.008 (−0.16 to −0.01)	<0.001
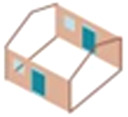	two large-screened windows	31.3 (31.2 to 31.4)	−0.41 (−0.50 to −0.32)	<0.001	29.1 (29.1 to 29.2)	−0.41 (−0.49 to −0.32)	<0.001
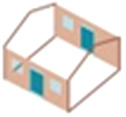	three large-screened windows	31.1 (31.1 to 31.2)	−0.55 (−0.64 to −0.46)	<0.001	29.0 (28.9 to 29.1)	−0.55 (−0.63 to −0.46)	<0.001

**Figure 3.**
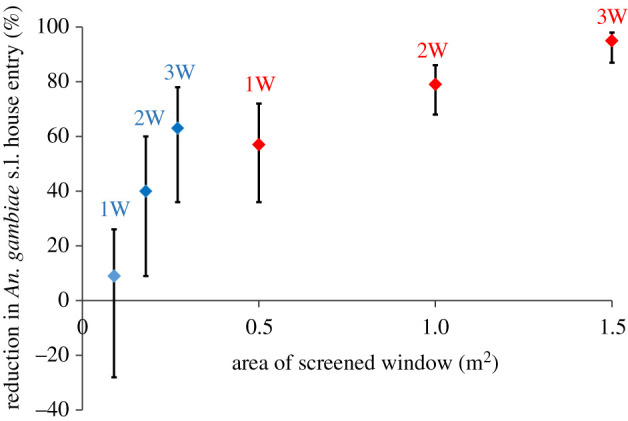
Relationship between the area of window screening and reduction in *An. gambiae* s.l. numbers indoors. Blue data points, small windows (experiment 1). Red data points, large windows (experiment 2). Reductions are relative to the reference house in each experiment. Error bars represent 95% confidence intervals. W refers to the number of windows in each house.

As the number of small- or large-screened windows increased, the number of *An. gambiae* s.l., and other mosquito species (electronic supplementary material, table S1), collected indoors decreased (table 2). A summary figure combining the results from experiment 1 and 2 shows the percentage of *An*. *gambiae* s.l. collected indoors declines with increasing number and area of the screened window, with two or three windows providing best protection (figure 3).

**Table 2.** Effect of screened windows and door gaps on house entry by *An. gambiae* s.l. General linearized modelling results, adjusting for house position and night. Data are means (95% CI). Larger drawings of the house typologies are shown in figure 1.

		screened window area (m^2^) (*n* = 25)	no. of *An. gambiae* s.l. per night (*n* = 25)	adjusted estimates, mean ratio	*p*-value
experiment 1: small-screened windows					
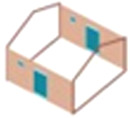	two badly fitting small windows (reference)	—	6.16 (4.07 to 8.25)	1.0	—
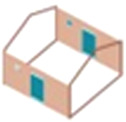	one small-screened window	0.09	6.44 (4.04 to 8.84)	0.91 (0.65 to 1.28)	0.586
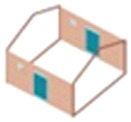	two small-screened windows	0.18	3.80 (1.15 to 6.45)	0.60 (0.40 to 0.91)	0.015
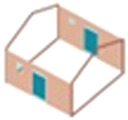	one small-screened window and one medium-screened window	0.27	2.60 (1.22 to 3.98)	0.37 (0.22 to 0.64)	<0.001
experiment 2: large-screened windows					
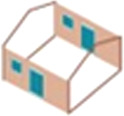	two badly fitting large windows (reference)	—	11.04 (7.72 to 14.36)	1.0	—
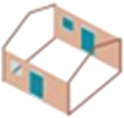	one large-screened window	0.50	4.72 (3.16 to 6.28)	0.43 (0.28 to 0.64)	<0.001
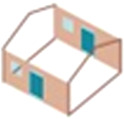	two large-screened windows	1.00	2.64 (1.55 to 3.73)	0.21 (0.14 to 0.32)	<0.001
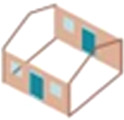	three large-screened windows	1.50	0.56 (0.15 to 0.97)	0.05 (0.02 to 0.10)	<0.001
experiment 3: door gaps					
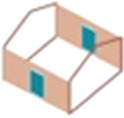	gap above and below the doors, no windows (reference)	—	2.04 (1.45 to 3.35)	1.0	—
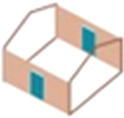	gaps above the doors, no windows	—	3.96 (2.43 to 5.49)	1.28 (0.83 to 1.97)	0.259
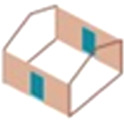	gap below the doors, no windows	—	3.28 (2.13 to 4.43)	1.30 (0.81 to 2.09)	0.285
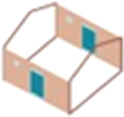	gaps above and below doors, with two small-screened windows	—	2.92 (1.52 to 4.32)	1.05 (0.63 to 1.76)	0.844

In experiment 3, reducing the number of gaps around the door from two to one or installing two small-screened windows into the house did not reduce the numbers of *An. gambiae* s.l. collected indoors compared with the reference house (table 2). Numbers of *Mansonia* spp. and *Culex* spp., however, were lower in houses where there was no gap at the bottom of the door, compared to the reference house with gaps both at the top and bottom of the door (electronic supplementary material, table S1).

**Figure 4.**
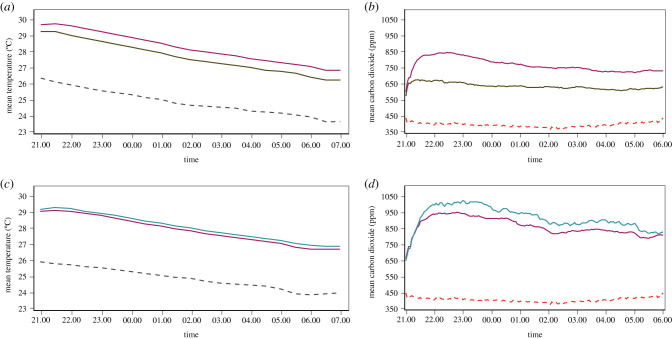
Nocturnal indoor and outdoor temperature (*a,c*) and indoor CO_2_ concentrations (*b*,*d*) in screened and unscreened houses. Magenta lines, houses with screened windows and solid doors. Green lines, houses with screened windows and doors. Blue solid lines, houses with no windows and solid doors. Black broken lines, outdoor temperature. Red broken lines, outdoor CO_2_ concentration. All measurements were made in year 2, apart from outdoor CO_2_, which was made in year 1.

In experiment 4, there were 76% fewer *An. gambiae* (95% CI 69–82%, *p* < 0.001) in houses with screened doors and screened windows than those with solid doors and screened windows. Temperatures in houses with screened doors were 0.6°C less than houses with screened doors before midnight (95% CI 0.4–0.7°C, *p* < 0.001) and after midnight (95% CI 0.5–0.7°C, *p* < 0.001). Indoor concentrations of CO_2_ rose above background levels shortly after two men entered each house at 21.00 h, to a maximum roughly 1 h later, before declining gradually through the night, before a small rise around 05.00 h, one hour before the men left the houses (figure 4). Indoor CO_2_ concentrations were 152 ppm (95% CI 109–195 ppm, *p* < 0.001) lower in screened door houses than those with solid doors from 19.00 to 23.59 h (*p* ≤ 0.001) and 120 ppm lower (95% CI 81–159 ppm, *p* ≤ 0.001) from 00.00 to 05.59 h.

In experiment 5, there were 38% (95% CI 23–50%, *p* < 0.001) fewer *An. gambiae* entering houses with two screened windows compared to control houses without windows. Temperatures in houses with two large-screened windows were 0.2°C cooler before midnight (95% CI 0.0–0.3°C, *p* = 0.019) and after midnight (95% CI 0.1–0.3°C, *p* < 0.001) than the reference house. In houses with screened windows, indoor CO_2_ concentrations were 81 ppm (95% CI 11–150 ppm, *p* = 0.027) lower than those without windows from 19.00 to 23.59 h and 83 ppm less (95% CI = −4 to 170 ppm, *p* = 0.060) from 00.00 to 06.00 h.

Also, in experiment 5, although CO_2_ concentrations did not rise with increasing temperature in houses with screened windows, they did in houses without. In houses with screened doors and windows, there were similar CO_2_ concentrations before midnight (45 ppm, 95% CI −2 to 91 ppm, *p* = 0.059) and after midnight (9 ppm, 95% CI −30 to 48 ppm, *p* = 0.626). Similarly, CO_2_ concentrations in houses with screened windows did not vary before (9 ppm, 95% CI −22 to 40 ppm, *p* = 0.555) and after midnight (6 ppm, 95% CI −31 to 43 ppm, *p* = 0.751). In marked contrast, for a 1°C rise in indoor temperature in unventilated houses, there was a 60 ppm increase in CO_2_ before midnight (95% CI 24–96 ppm, *p* = 0.004) and 73 ppm (95% CI 43–104 ppm, *p* < 0.001) after midnight.

### 3.2. Computational fluid dynamic modelling

**Figure 5.**
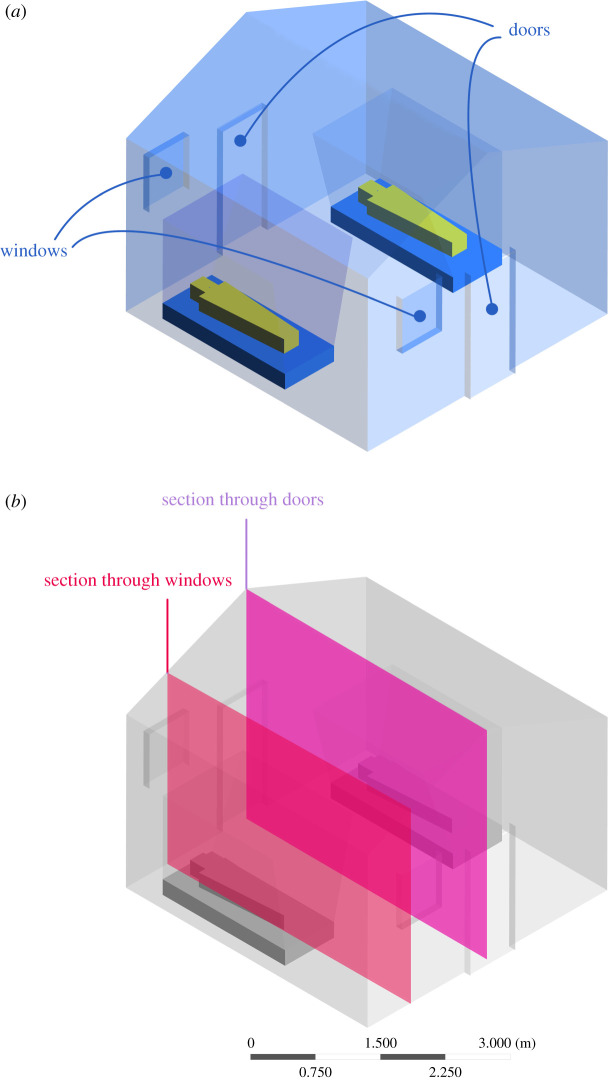
(*a*) Structure of an experimental house showing (*b*) sections through windows and doors. Here, two men sleep under a bednet in a house with two badly fitting doors and two screened windows. The illustration represents the house typology common in experiments 4 and 5 (figure 1). The sections taken here are used to show CO_2_ concentrations in figures 6 and 7.

**Figure 6.**
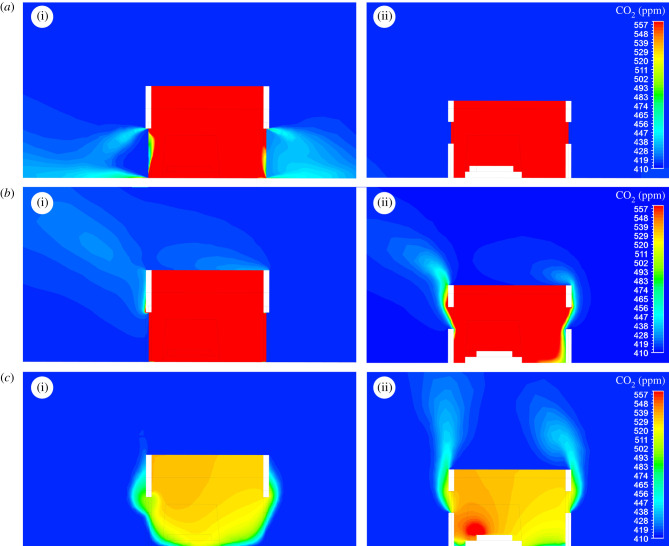
CFD simulations of CO_2_ produced from two sleeping adults in unscreened and screened houses with two badly fitting doors in windless conditions: (*a*) houses without screened doors or windows; (*b*) screened windows; (*c*) both screened windows and doors. (i) and (ii) indicate the section is through door or windows, respectively (figure 5). Note that CO_2_ is released from narrow gaps above and below all doors and windows, and through screening. In (*a*)ii, (*b*)ii and (*c*)ii the section through the house includes a sleeping person generating CO_2_ from the head. Note that the legend limits were chosen to enable comparisons between the different typologies and that the maximum value of 557 ppm of CO_2_ in the legend was exceeded in simulations (*a*) and (*b*), with maximum values of over 1000 ppm; typically around the mouths of the sleepers.

**Figure 7.**
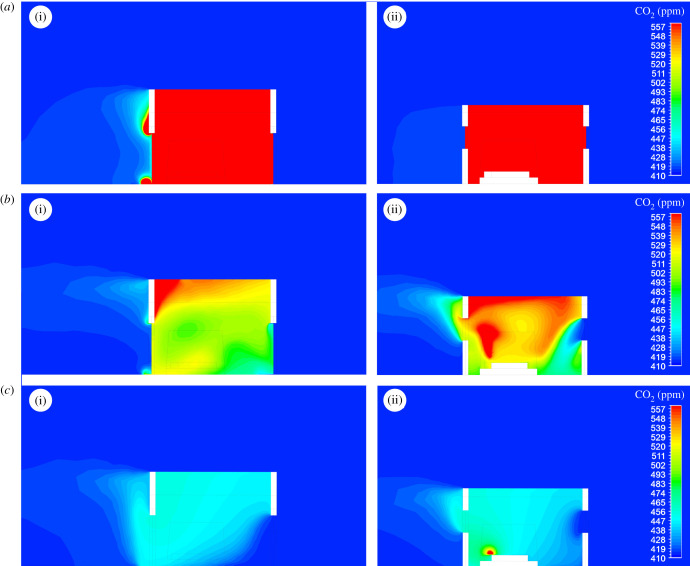
CFD simulations of CO­_2_ produced from two sleeping adults in unscreened and screened houses with two badly fitting doors in field conditions where the average wind speed is 0.44 m s^−1^: (*a*) houses without screens; (*b*) screened windows; (*c*) both screened windows and doors; (i) and (ii) indicate the section is through door or windows, respectively (figure 5). Note that CO_2_ is released from narrow gaps above and below all doors and windows, and through screening. In (*a*)ii, (*b*)ii and (*c*)ii, the section through the house includes a sleeping person generating CO_2_ from the head. Note that the legend limits were chosen to enable comparisons between the different typologies and that the maximum value of 557 ppm of CO_2_ in the legend was exceeded in simulations (*a*), with maximum values of over 1000 ppm; typically around the mouths of the sleepers.

The exchange of CO_2_ between the indoor and outdoor environment of a house is shown through sections made through the doors and windows of the experimental houses (figure 5). The flow of CO_2_ out of a house differs according to whether doors and windows are screened and whether there is no wind (figure 6) or light wind (figure 7). With no wind, unscreened houses produce horizontal jets of CO_2_ from gaps below and above the doors reaching more than 4 m from a house. When screened windows are installed, little CO_2_ is streamed from the doors, with most leaking from the windows, rising vertically into space. In houses with screened doors and windows, less CO_2_ accumulates indoors and low levels are projected from the doors and windows, rising upwards. On windy nights, screening reduces the concentration of CO_2_ indoors (figure 7). On the windward side of the house, little or no CO_2_ escapes from all house typologies. In marked contrast, on the leeward side, high concentrations of CO_2_ leak from above and below the door of unscreened houses. Screening results in markedly less CO_2_ leaking from the screened doors and windows, presenting a diffuse source of the gas.

## 4. Conclusion

Well-ventilated houses are not only cooler at night and more comfortable to live in, they also have markedly fewer malaria mosquitoes than poorly ventilated houses. Installing two or more large-screened windows, each 0.5 m^2^ in area, lowered the indoor temperature at night by 0.2–0.6°C, depending on outdoor wind speed. Adding two screened doors to a house with screened windows further reduced the temperature. Cross-ventilation of the room, where windows are positioned on opposite walls, without any internal obstruction, is essential to encourage airflow and reduce the indoor temperature. Increased ventilation makes it more comfortable for the occupants before midnight, making it more likely they will use an ITN. By contrast, after midnight, houses with screened windows or doors cool down further and become uncomfortably cool. Being too cool at night, however, is easily resolved by sleeping under a sheet, while being too hot, is impossible to resolve without fans, air conditioning or sleeping under a wet towel or sheet. The cooling effect of screening is also supported by our previous findings in The Gambia and elsewhere [9]. Nonetheless, the cooling effect of cross-ventilation is limited in the hot humid tropics, especially where using a bednet will reduce airflow by an average of 64% [20].

Surprisingly, we discovered that increasing both the number and size of screened windows in a Gambian house reduced house entry of *An. gambiae* s.l. mosquitoes. With three screened windows and a total surface area of 1.5 m^2^, there was a 95% reduction in the number of *An. gambiae* s.l. found indoors. This level of protection against malaria vectors is equivalent to that seen with some ITNs in the same area [21], but in our case, no insecticides were used on the screening or doors. The only source of insecticide was on the ITNs. Similar findings were observed with other taxa of mosquitoes, suggesting that the addition of screening can reduce the house entry of many species of African mosquitoes. While it is well known that a screened house provides a physical barrier against mosquitoes, this study is the first to show screening reduces mosquito entry in houses which are not perfectly closed and are therefore more representative of typical village houses.

We explored three hypotheses to explain the indirect protective effect of screening. First, our findings suggest that mosquitoes are not attracted in large numbers to external faces of screening, since houses with slits above and below screened doors had markedly fewer, not more, mosquitoes than those with solid doors and slits. Second, and more plausibly, the mechanism of protection results from screening reducing the concentration of CO_2_ dispersing from a house. CO_2_ concentrations were approximately 80 ppm less in houses with screened windows compared to houses without windows and adding two screened doors to a house with screened windows resulted in a further 120–152 ppm reduction. Houses with screened doors will leak CO_2_ more rapidly than the door gaps common in most rural houses. The reductions in CO_2_ levels are of biological significance since female mosquitoes can detect differences in CO_2_ concentration as small as 40 ppm [22]. Interestingly, in poorly ventilated houses, CO_2_ concentration increased with increasing indoor temperature. This is probably related to higher rates of metabolism associated with sweating and keeping the body cool. Our third hypothesis about the structure of the odour plume emanating from the house is also likely to be important. Computer simulations show that jets of CO_2_ project from unscreened houses from above and below the doors, both accessible entry points for malaria mosquitoes. In marked contrast, in screened houses, the odour plume is weaker and rises upwards. Thus blood-seeking mosquitoes are likely to detect an unscreened house from greater distances than a screened house, potentially explaining why more mosquitoes find their way into an unscreened house compared to a screened one. In nature, the picture is more complicated, since a blood-seeking mosquito finds itself in an environment where there is a continuous plume of CO_2_ from a house, when there is no wind, and turbulent pulses of CO_2_, when the wind blows and eddies around the house. *Anopheles gambiae* is, however, able to orientate readily along such turbulent odour plumes, moving towards the odour source.

There are four main limitations to this study. First, the experiment was carried out without considering the variability in ‘real world’ human behaviour. The sleepers went to bed earlier than many adults, the doors remained closed during the night, unlike those in the villages which are frequently opened and closed until midnight [11], and there was no wood smoke or bright lights indoors. Second, while our focus is on CO_2_ as a long-distance attractant, there are other volatiles produced by humans that are also strongly attractive to *An. gambiae* s.l. [23,24]. We do not know how these short-distance attractants affect mosquito movement in our study. Thirdly, there were only two men in each house, fewer individuals than most village houses. Increasing the number of people sleeping indoors is likely to increase CO_2_ concentrations, and hence indoor mosquito collections [25]. Fourth, our study used single-roomed houses, and the effects reported here are likely to vary according to different house typologies including those with multiple rooms, ceilings, porosity of structure, and presence and proximity of adjacent buildings.

Findings from the present study suggest that screening is not only an effective barrier against mosquitoes, it also increases ventilation, cools the house and reduces CO_2_ concentration indoors. In houses with screening, the CO_2_-odour plumes released from the house are likely to attract mosquitoes from shorter distances, reducing the number that enter the house, compared to unscreened houses with badly fitting doors. The World Health Organization recommends house improvements, such as installing window screens, for reducing malaria [26,27]. There has never been a better time for developing new ways of screening houses and facilitating the scale-up of this intervention, since it coincides with a period when Africa's housing stock is modernizing [28], and more housing is urgently needed to accommodate the more than one billion new Africans that will need homes in rural areas by 2050 [29]. Building healthy homes that reduce the threat from malaria and other mosquito-borne diseases, that prevent over-crowding, increase airflow, reduce indoor air pollution, and keep the occupants clean, secure and comfortable during the day and night is an imperative and a basic human right.

## Data accessibility

All data used in this paper are available as electronic supplementary material.
